# Clinicopathologic importance of atypical glandular cells in cervico-vaginal cytology

**DOI:** 10.4274/jtgga.galenos.2019.2019.0059

**Published:** 2020-06-08

**Authors:** Seda Yüksel, Erhan Şimşek, Selçuk Yetkinel, Songül Alemdaroğlu, Filiz Aka Bolat, Hüsnü Çelik

**Affiliations:** 1 Clinic of Obstetrics and Gynecology, Başkent University, Adana Dr. Turgut Noyan Practice and Research Center, Adana, Turkey

**Keywords:** Cervical cancer, neoplasms, pap smear

## Abstract

**Objective:**

To analyze the histopathologic outcomes of patients with atypical glandular cells (AGC) in cervicovaginal cytology examinations.

**Material and Methods:**

Patients with AGC in cervicovaginal cytology were included in this study between March 2011 and March 2018 and patient data were collected retrospectively among all cytology results. AGC classification of cervicovaginal cytology were based on the Bethesda 2001 classification system.

**Results:**

The total prevalence of cervical epithelial cell abnormality and AGC were found as 4.2% and 0.2%, respectively, in the study cohort. AGC-favor neoplasia (AGC-FN) was the subgroup of AGC with the highest malignancy rate with 62.5% (p=0.06). The incidence of malignancy in the postmenopausal group (33.3%) was detected higher than in the premenopausal group (8.3%) (p=0.07).

**Conclusion:**

The probability of malignancy in AGC-FN cytology is more commonly associated with malignancy in the postmenopausal group. Therefore, histopathologic examination is strongly recommended in these patients with AGC smears because of the high risk for malignancy in this group.

## Introduction

Preinvasive lesions of the cervix can be diagnosed with Papanicolaou smear tests and be treated long before overt carcinoma develops. Routine cervical cancer screening programs in many countries significantly reduced the incidence and mortality rate of cervical cancer (1). A thorough understanding of cervical cancer pathogenesis and the development of effective screening programs both with cervical cytology and human papilloma virus (HPV) typing and vaccination against high-risk HPV types have significantly altered the distribution of cervical cancer and premalignant lesions of the cervix in countries where screening programs cover the majority of the population. Although the incidence of squamous cell cancers of the cervix is decreasing, the rate of adenocarcinomas among cervical cancers is either unchanged or increasing (2). There are many reasons for this relative increase of cervical adenocarcinoma. First, the location of adenocarcinoma and its preinvasive lesion; adenocarcinoma in situ (AIS) is rather deep and with higher localization within the cervical crypts, which makes these lesions difficult to recognize, like their squamous counterpart lesions. Second, cytologic and colposcopic signs of AIS lesions are not easy to recognize, as with squamous pathologies. Thirdly, invasive adenocarcinomas may originate from a small foci of AIS areas of the cervix (3).

Glandular cell anomalies in cervical cytology are relatively rare compared to squamous cell anomalies. The incidence of atypical glandular cell (AGC) was reported as 0.17% in a recent large study on cervical cytologic screening (4). In another study in a tertiary referral center, the incidence of squamous and glandular abnormalities were found as 1.5% and 0.4%, respectively (5). Another population-based study including patients with AGC cytology reported a 1.4% risk for developing invasive cervical carcinoma, whereas this risk was found as 2.5% and 0.2% in patients with high-grade intraepithelial lesion and low-grade squamous intraepithelial lesion cytology, respectively (1).

In the current literature, AGC is questioned to be associated with severe cervical pathologies. Atypical glandular cervicovaginal cytologic abnormalities are more frequently associated with cervical adenocancer, AIS and cervical squamous lesions than squamous cervicovaginal cytologic abnormalities.

In this study, we aimed to analyze the relationship between cervicovaginal cytologic glandular abnormalities with cervical malignant pathologies. For this purpose, cervicovaginal cytology reports were examined between March 2011 and March 2018, retrospectively, and histopathologic surveillance of patients who were diagnosed as having AGCs were analyzed and resultant cervical malignancies have been traced.

## Material and Methods

Liquid-based (ThinPrep Pap Test, Hologic) cervicovaginal cytologic examinations that were performed between March 2011 and March 2018 within the context of an opportunistic cervical screening program were reviewed, and the patients reported as having AGCs were detected. The diagnostic and pathologic examinations following the cytologic examinations in these patients were obtained retrospectively by reviewing the patients’ medical records. All cytology and pathology specimens were re-evaluated by the department of medical pathology as needed. The Bethesda 2001 classification system was used to classify the AGCs. The Bethesda 2001 system classifies AGCs as follows: AGC-not otherwise specified (AGC-NOS), AGC-endocervical cells (AGC-EC), AGC-endometrial cells (AGC-EM), and AGC-favor neoplasia (AGC-FN). Only cytologies obtained from cervix uteri were included in this study. AGC results of vaginal cuff cytologies were excluded. The results of these groups are explained separately.

This retrospective study was approved by the institutional ethical committee (approval number: KA18/230).

### Statistical analysis

The SPSS 17.0 (IBM, USA) software was used for statistical analysis. All independent parameters were analyzed using the chi-square test and the Mann-Whitney U test. P values <0.05 were accepted as statistically significant.

## Results

**Figure 1:**
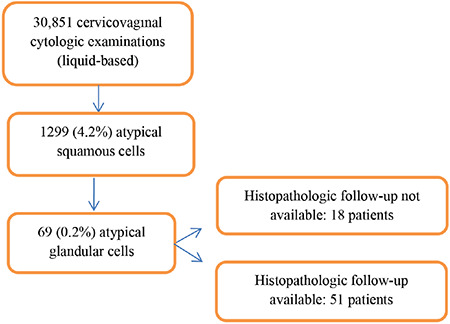
Flow diagram of the study

It was determined in the study that a total of 30,851 cervicovaginal cytologic examinations were performed between March 2011 and March 2018. Epithelial cell abnormality was encountered in 1299 patients (4.2%), and AGCs were detected in 69 patients (0.2%) (Figure 1). Cytology was obtained from the vaginal cuff in 17 of 69 patients. Fourteen of these 17 patients were diagnosed as having endometrial cancer and underwent surgery. During surveillance, AGCs were detected and further histopathologic examinations were performed. As a result, three cases of recurrence were found. There was one patient with cervical cancer and two patients underwent hysterectomy with benign indications. These 17 patients were excluded because the cytologic materials were obtained from the vaginal cuff, and majority of the patients were already diagnosed as having gynecologic malignancy.

The median age of the patients with AGCs was 47 (minimum: 25, maximum: 77) years, and 42.3% of patients (n=22) were postmenopausal. Sixty-five percent (n=34) of patients with AGCs were asymptomatic and were detected in routine cervicovaginal cytologic examinations, whereas symptoms of menometrorrhagia, menorrhagia, vaginal itching, urinary incontinence, postmenopausal bleeding, and leucorrhoea were reported in 6, 4, 1, 2, 2, and 3 patients, respectively. Further pathologic examinations were offered to all patients; 19% (n=10) were lost to follow-up and 80.7% (n=42) underwent histopathologic examinations with materials taken from the cervix, endocervical canal, and endometrial cavity as indicated.

The evaluation based on subtypes of AGC revealed AGC-NOS, AGC-EC, AGC-EM, and AGC-FN in 17 (32.6%), 23 (44.2%), 2 (3.8%), and 10 (19.2%) of patients, respectively (Table 1). Menopausal status was shown to be associated with the subtype distribution of AGCs in our study. AGC-EC was predominantly found in the premenopausal group (63%), whereas AGC-NOS (50%) was higher in the postmenopausal group, and these differences were found to be statistically significant (p=0.01). HPV genotyping was possible after 2016 and HPV status was examined in only 10 out of 52 patients; all patients were HPV negative except for one patient with low-risk HPV positivity.

Twenty-eight percent (n=12) of the 42 patients with available pathologic follow-up data were normal, whereas active chronic inflammation, CIN1, CIN3, cervical squamous cell carcinoma, cervical adenocarcinoma, endometrial mixed carcinoma, endometrial polyp, endometrial hyperplasia, metastatic carcinoma, and ovarian serous carcinoma were encountered in 10 (23.8%), 6 (14.2%), 2 (4.7%), 3 (7.1%), 2 (4.7%), 1 (2.3%), 3 (7.1%), 1 (2.3%), 1 (2.3%), and 1 (2.3%) patient, respectively.

Regarding the patients with a malignant final diagnosis, AGC-FN (62.5%) was shown to be by far the most frequent AGC diagnosis (p=0.06). The subtypes of AGCs in the patient group with malignant lesions according to the pathologic follow-up examinations were found to account for 50% of the entire AGC-FN group (Table 2). On the other hand, 66% of all malignant cytologies in postmenopausal patients were AGC-FN initially (p=0.1) (Table 3). No statistically significant difference was found between the subtypes of AGCs in patients with malignant pathologies in the premenopausal group (p=0.3) (Table 4).

In terms of menopausal status, the incidence malignancy in the postmenopausal group (33.3%) was higher than in the premenopausal group (8.3%) (p=0.07).

## Discussion

This study confirms that AGC is a rare cervico-vaginal cytologic abnormality with a prevalence of 0.2% out of 30,851 cytologic investigation. Similar prevalence rates of AGC have been reported in the literature (4,6,7,8). The prevalence of cervical malignant lesions within AGC cytology was 9.6%, whereas this rate reached 15.3% with the addition of all types of gynecologic malignancies, and 32.6% with the inclusion of premalignant lesions. The prevalence rates of the underlying neoplasia ranged between 9-50% according to AGC cytology reports, as in the literature (6,9). Tam et al. (10) reported that the risk for premalignant-malignant lesions in AGC-NOS cytology was 19%, whereas this risk rate was detected as 68% in the AGC-FN group (8). In our study, malignancy was encountered in 5 (50%) of the 10 patients with AGC-FN. When premalignant lesions were encountered, this rate was nearly 70% among patients with AGC-FN cytology. Among patients with a malignant final pathology, the leading prior AGC subtype was also AGC-FN in this cohort; nonetheless, the difference did not reach statistical significance (p=0.06).

AGC cytology may be due to cervical pathologies or endometrial pathologies. In one study on 41 patients with AGC cytology, endometrial cancer was detected in 13 patients, all of whom were aged over 40 years. It was reported in another study that endometrial pathology was especially found in patients aged over 45 years (11). It was suggested after the Bethesda 2014 revision that reporting age of patients with AGC-EM in cervical cytology should be adjusted as 45 years and over. This regulation was attributed to the presentation of endometrial pathologies, especially in the postmenopausal group (12). In this study, endometrial malignant pathology was encountered in one patient with endometrial mixed (serous + endometrioid) tumor; the age of this patient was 68 years. Benign pathologies of the endometrium included endometrial polyps and endometrial hyperplasia in three patients and one patient, respectively.

Our study showed that menopausal state was an important risk factor for AGC smears resulting with a final diagnosis of malignancy. Only two premenopausal patients (6.6%) were diagnosed as having malignancy, both of whom had cervical adenocarcinoma. The ages of these patients were 36 and 40 years. On the other hand, postmenopausal patients’ pathologic follow-up examinations showed that considerably more patients in the postmenopausal group were diagnosed as having malignancies (33.3%) as final diagnoses (p=0.07). Although this difference seems insignificant statistically, this may result from the limited number of patients. Therefore, AGC diagnoses in menopause must be evaluated cautiously because of the increased risk of a malignant tumors.

HPV co-testing with cervical cytology has an important role in the triage of cervical squamous lesions; however, its importance is not as clear in glandular pathologies and cervical adenocancer. In a Swedish population-based study, it was reported that the HPV reflex test had a very positive predictive value in the prediction of high-grade cervical lesions in patients with AGCs, and that the planning of a follow-up schedule based on HPV status would be reasonable (13). A systematic review that analyzed the importance of HPV in AGC cytology noted that the hr-HPV test had a sensitivity of 90% in the prediction of CIN-2 and higher lesions in patients with AGC (14). In our study, only a minority of the patients diagnosed as having AGCs had co-testing with HPV because we perform colposcopy to all patients with AGCs; the absence of HPV co-testing was not a concern other than for selecting patients who could be followed up less often if their HPV test were found as negative. HPV status was analyzed in only 10 patients in our study group and 9 patients were found as HPV-negative, whereas one patient had low-risk HPV positivity. No interpretation could be made about the importance of HPV in the triage of AGC because HPV status was unknown for all 52 patients with AGCs in our study.

The guideline of the American Society for Colposcopy and Cervical Pathology has recommended colposcopy and endocervical sampling in the management of AGC (15). In accordance with this guideline, we also perform colposcopic examinations, and cervical and endocervical sampling in patients.

There is more debate as to which patients should undergo endometrial biopsy. Endometrial sampling can be recommended according to the age and symptoms of the patient. Although AGC subtype with AGC-EM constitutes only a minority of AGC cytologies, this sub-group carries higher risk for endometrial pathologies and malignancies. The small number of patients and also the retrospective nature of our cohort precludes us from drawing firm conclusions.

## Conclusion

The detection of AGCs on cervicovaginal cytology carries a potential risk of various malignancies, particularly in postmenopausal patients. It can be stated from our study that among all AGC subtypes, AGC-FN cytologies are more commonly correlated with malignancy and this risk was particularly high for postmenopausal patients. Any result with AGCs necessitates further investigation with histopathologic examination. Future studies with large patient series on AGCs at cervicovaginal cytology may help to delineate patients at risk for malignancies.

There is no conflict interest between the authors of the manuscript.

## Figures and Tables

**Table 1:**
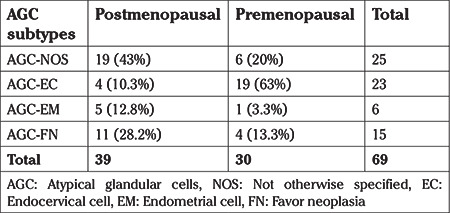
Subtype distribution of atypical glandular cells based on menopausal status

**Table 2:**
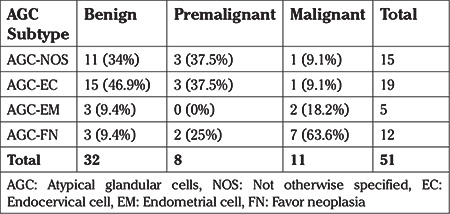
The distribution of histopathologic results according to subtype of atypical glandular cells

**Table 3:**
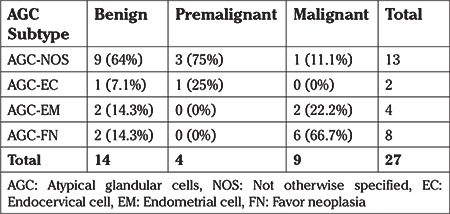
The distribution of histopathologic results according to subtype of atypical glandular cells in the postmenopausal group

**Table 4:**
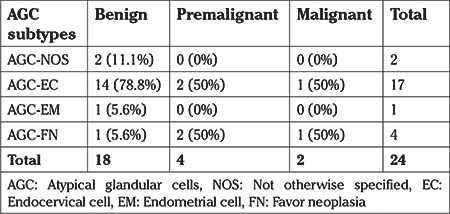
The distribution of histopathologic results according to subtype of atypical glandular cells in the premenopausal group
